# Ultrasound staging and fine needle aspiration cytology: how well do they predict breast cancer nodal involvement?

**DOI:** 10.1186/bcr2960

**Published:** 2011-11-04

**Authors:** JM Schneider, A Leaver

**Affiliations:** 1 University of Dundee, UK; 2 Gateshead Health NHS Foundation Trust, Gateshead, UK

## Introduction

Ultrasound and fine needle aspiration cytology (FNAC) are used to provide presurgical axillary assessment in breast cancer, in line with NICE guidance. An N1 to N5 ultrasound staging process has been recently introduced. The significance of the new N staging system and overall preoperative staging results has been analysed.

## Methods

Patient data were collected during MDTs and from the electronic results and analysed retrospectively. Patients with ultrasound score N and/or axillary FNAC were included if they had subsequent histology (sentinel lymph node biopsy or axillary clearance).

## Results

A total of 125 patients had histological node samples following ultrasound assessment and/or FNAC. Fifty-eight had both ultrasound and FNAC. Ultrasound/FNAC were found to be 82% (27/33)/79% (23/29) sensitive and 100% (44/44)/100% (41/41) specific, respectively. The overall preoperative staging process sensitivity was 65%. The positive predictive values of N3, N4, and N5 were 31% (10/32), 50% (6/12) and 100% (11/11), respectively.

## Conclusion

Combining ultrasound and FNAC assessment provides a valuable method of preoperative lymph node staging, guiding surgical management, reducing unnecessary surgery and the number of repeat operations. Our ultrasound N staging system correlates well with final histology, and we now plan to introduce repeat axillary biopsy with high stage N/negative FNAC patients, to attempt an overall increase in preoperative staging sensitivity/a more effective management pathway.

